# Editorial: Human-Interpretable Machine Learning

**DOI:** 10.3389/fdata.2022.956625

**Published:** 2022-06-20

**Authors:** Gabriele Tolomei, Fabio Pinelli, Fabrizio Silvestri

**Affiliations:** ^1^Department of Computer Science, Sapienza University of Rome, Rome, Italy; ^2^Systems Modeling and Analysis Research Unit, IMT School for Advanced Studies Lucca, Lucca, Italy; ^3^Department of Computer Engineering, Sapienza University of Rome, Rome, Italy

**Keywords:** explainable AI (XAI), machine learning, counterfactual explanations, time-series classification (TSC), adversarial learning, rule extraction, computer vision

This Research Topic encouraged submissions that broadly address the challenge of making machine learning (ML) models more transparent and intelligible to humans. Indeed, this follows the spirit of the *explainable AI* (XAI) initiative (Samek et al., 2019), which promotes efforts to improve the human-interpretability of ML systems, especially those supporting decisions in social domains like finance (Bracke et al., 2019) and healthcare (Ahmad et al., 2018).

In this Research Topic, Guidotti and D'Onofrio propose MAPIC, a novel and efficient method to train time-series classification models that are natively interpretable by design based on *matrix profiles* (i.e., roughly, distances between all the time-series subsequences and their nearest neighbors). Time-series classification is a pervasive and transversal problem in various domains, ranging from disease diagnosis to anomaly detection in finance. Inspired by previous work on time-series classifiers based on *shapelets* (Ye and Keogh, 2009; Trasarti et al., 2011), MAPIC operates as follows. First, to find the best shapelets, MAPIC exploits the matrix profiles extracted from the time-series of the training set instead of using a brute force approach (Ye and Keogh, 2009) or an optimized search (Grabocka et al., 2014). Hence, MAPIC retrieves motifs and discords from the matrix profiles of each time-series and adopts them as candidate shapelets. Second, differently from traditional approaches that learn machine learning models for time-series classification directly on all the shapelet transformations (Grabocka et al., 2014), MAPIC builds a decision tree by refining at each splitting point the set of candidate shapelets that better represent the times-series in the current split. Experimental results demonstrate that MAPIC outperforms existing approaches with similar interpretability in accuracy and running time.

Abbasi-Asl and Yu, instead, focus their attention on computer vision and introduce a greedy structural compression method to obtain smaller and more interpretable CNNs while achieving close to original accuracy. The compression scheme proposed operates by pruning filters in CNNs. Authors define a filter importance score to select candidate filters to be discarded, which corresponds to the network's classification accuracy reduction (CAR) after pruning that filter. Furthermore, the authors show the ability of their proposed technique to remove functionally redundant filters, such as color filters, making the compressed CNN's more accessible to human interpreters without much classification accuracy loss. Interestingly enough, the advantages of this method go beyond explainability as the structural compression of the network architecture also allows a space-efficient deployment of the model on resource-constrained devices.

Tang et al. approach the explainability issue from a different yet related perspective, namely adversarial learning. As shown recently, explanation methods are vulnerable to adversarial manipulation. Ghorbani et al. (2019) show that one can change the model's explanation while keeping its prediction fixed. To tackle this problem, the authors propose a new training methodology called Adversarial Training on EXplanations (ATEX) to improve the internal explanation stability of a model regardless of the specific explanation method used. Instead of directly specifying explanation values over data instances, ATEX only puts constraints on model predictions, avoiding involving second-order derivatives in the optimization process. Moreover, the authors find that explanation stability is closely related to model robustness, i.e., resiliency to adversarial attacks. Experiments demonstrate that ATEX improves model robustness against the manipulation of explanations, and they also show that ATEX increased the efficacy of adversarial training. Overall, this study confirms the strong relationship between adversarial attack robustness and interpretation, which is a promising line of future research work.

Finally, Vilone and Longo propose a novel comparative approach to evaluate the rule sets produced by five *post-hoc* explanation methods, i.e., C45Rule-PANE (Zhou and Jiang, 2003), REFNE (Zhou et al., 2003), RxNCM (Biswas et al., 2017), RxREN (Augasta and Kathirvalavakumar, 2012), and TREPAN (Craven and Shavlik, 1994), which are all designed to extract rules from black-box feed-forward neural networks. Authors manually trained these models on 15 datasets with handcrafted features engineered by humans. The authors use eight validity metrics proposed in the literature to assess the degree of explainability of the rule sets extracted by the five explanation methods: the ruleset cardinality, the number of antecedents, completeness, fidelity, correctness, and robustness, and the fraction of classes and overlap. The authors run a Friedman test (Friedman, 1937) to determine whether a method consistently performs better than the others in terms of the selected metrics and could be considered the best-performing one overall. Findings demonstrate that there is no sufficient evidence to identify one superior method over the others. These validity metrics capture distinct aspects of explainability, providing vital insights into what a model has learned during its training process and how it makes its predictions.

As decisions taken by intelligent systems will increasingly impact many aspects of our daily lives, the demand for explainable AI/ML models will become even more prominent. The large body of work on this subject in recent years, e.g., Tolomei et al. (2017), Tolomei and Silvestri (2021), Lucic et al. (2022), and Siciliano et al. (2022), testifies its importance. Also. the effort that government organizations like the European Union have put into this (see Article 22 of EU's General Data Protection Regulation EU, 2016) shows the importance of such a topic.

On the one hand, we hope that this Research Topic represents a valuable reference for those approaching the subject of explainable AI/ML for the first time. In addition, we wish to inspire readers who are already familiar with AI/ML explainability with the contributions of this Research Topic to propose new explanation methods and techniques, thus pushing forward the state-of-the-art knowledge on *Human-Interpretable Machine Learning*.

## Author Contributions

All authors listed have made a substantial, direct, and intellectual contribution to the work and approved it for publication.

## Funding

This research was supported by the Italian Ministry of Education, University and Research (MIUR) under the grant Dipartimenti di eccellenza 2018-2022 of the Department of Computer Science and the Department of Computer Engineering at Sapienza University of Rome.

## Author Disclaimer

All content represents the opinion of the authors, which is not necessarily shared or endorsed by their respective employers and/or sponsors.

## Conflict of Interest

The authors declare that the research was conducted in the absence of any commercial or financial relationships that could be construed as a potential conflict of interest.

## Publisher's Note

All claims expressed in this article are solely those of the authors and do not necessarily represent those of their affiliated organizations, or those of the publisher, the editors and the reviewers. Any product that may be evaluated in this article, or claim that may be made by its manufacturer, is not guaranteed or endorsed by the publisher.
